# Incidence of celiac disease autoimmunity and associations with maternal tuberculosis and pediatric *Helicobacter pylori* infections in 4-year-old Ethiopian children followed up in an HLA genotyped birth cohort

**DOI:** 10.3389/fped.2022.999287

**Published:** 2022-10-26

**Authors:** Adugna Negussie Gudeta, Carin Andrén Aronsson, Bayissa Bekele Binagdie, Alemayehu Girma, Daniel Agardh

**Affiliations:** ^1^ Unit of Diabetes and Celiac Disease, Department of Clinical Sciences, Clinical Research Center, Lund University, Malmö, Sweden; ^2^ Adama Public Health Referral and Research Laboratory Center, Adama, Ethiopia; ^3^ Department of Pediatrics, Adama Hospital Medical College, Adama, Ethiopia

**Keywords:** autoimmunity, celiac disease, children, cohort, Ethiopia, incidence, HLA

## Abstract

**Background:**

The prevalence of celiac disease in the general population is mainly unknown in most of sub-Saharan African countries. The aim of this study was to determine the incidence of celiac disease autoimmunity (CDA) and its associations with latent *Mycobacterium tuberculosis* (LMTB) and *Helicobacter pylori* (HP) infections in Ethiopian children aged 4 years in an HLA genotyped cohort study.

**Methods:**

Of 1,389 recruited children between 2018 and 2022, 1,046 (75.3%) had been screened at least twice for celiac disease between the ages of 2 and 4 years using a tissue transglutaminase autoantibody (tTGA) ELISA kit. Tissue TGA-positive children were retested using radio-binding assays. CDA was defined as persistent-confirmed tTGA positivity in two consecutive samples. Associations of CDA with LMTB and HP were tested in a subpopulation of 752 children born to mothers who were previously tested for LMTB with IFN-*γ* and anti-HP antibodies in samples collected at a mean age of 49.3 ± 5.3 months.

**Results:**

Screening detected 38 out of 1,046 (3.6%) IgA-tTGA-positive children. Ten (1.0%) were confirmed to be positive, with six (0.6%) children diagnosed with CDA. The incidence of CDA at 4 years of age was 1.2 per 1,000 person-years. LMTB was found in 4 of 6 (66.7%) mothers with CDA children compared with 340 of 734 (46.3%) mothers of children without CDA (*p* = 0.424), while HP was found in 3 of 6 (50.0%) CDA children compared with 315 of 746 (42.2%) children without CDA (*p* = 0.702).

**Conclusion:**

The incidence of CDA in Ethiopian children is lower than the pooled global incidence. Neither LMTB nor HP infections are associated with CD in Ethiopian children.

## Introduction

Celiac disease (CD) prevalence varies globally depending on the distribution of the HLA-DQ2 and HLA-DQ8 haplotypes and wheat consumption in the general population (1). The increase in incidence observed in many countries over the past few decades can be attributed to either increased awareness of the disease or as a result of changes in the environment (2). However, due to limited access to diagnostic testing and the diverse clinical manifestation, CD is most likely underestimated in many developing countries (1, 3). Serological tests are widely used in the diagnosis of CD. Tissue transglutaminase autoantibody (tTGA) is considered the best serological screening marker for CD since it has high sensitivity (78%–100%) and specificity (90%–100%) (4) and tTGA levels correlate with intestinal mucosal damage (5).

CD may develop at any age but has a peak incidence occurring in early childhood and a second peak in the third decade of life (6). Similar to many other autoimmune diseases, females are predominantly affected (7, 8). CD has a wide range of symptoms that make the disease difficult to diagnose in a clinical setting. In fact, population-based studies show that the majority of individuals with CD are diagnosed only through screenings (9). Despite the fact that the global prevalence of CD is believed to be around 1.4% (1), the true frequency of CD is mainly unknown in most countries around the world (10).

Although environmental factors like gluten and HLA-DQ2 or DQ8 are associated with CD, the actual etiology of CD is still unknown (11). The rising incidence supports the role of environmental risk factors, including exposure to microorganisms that may cause an inflammatory response in the small intestine. Latent *Mycobacterium tuberculosis* (LMTB) and *Helicobacter pylori* (HP) are common bacterial infections that affect humans globally. The occurrences of these two chronic infections are higher in developing countries, countries with low socioeconomic wealth, and in densely populated areas. There are numerous ways in which autoimmune diseases are brought on by pathogenic microorganisms. The molecular mimicry between microbial antigens and host proteins is one of the etiological paradoxes in the onset of autoimmune diseases. Studies indicate that TB-reactive T-cells can potentially be recognized as self-antigen, and this cross-reactive epitope may trigger autoimmune responses. A number of previous studies have shown that rheumatoid arthritis, multiple sclerosis, and other chronic inflammatory diseases are widespread in tuberculosis-endemic areas (12). On the other hand, studies from Karelia in Russia and Finland indicate that people living in deprived socioeconomic situations and lower hygienic conditions are at a lower risk of type 1 diabetes and CD, supporting the hygiene hypothesis that exposures to chronic infections and parasites may be protective from autoimmune diseases (13).

Ethiopia is one of the few endemic countries in the world where both HP and LMTB are two highly prevalent and clinically silent infections, whereas CD is still considered a rare disease (14, 15). Several previous studies on LMTB and HP have confirmed a prevalence as high as 50% for both infections in different regions of the country (16, 17). In a recent study on adult Ethiopian woman, we found a 0.05% seroprevalence of celiac disease autoimmunity (CDA) (15). It is hypothesized that differences in prevalence could indicate that low-grade infections might protect from autoimmune diseases, further supporting the hygiene hypothesis as demonstrated in the Russian Karelian and Finnish studies (13). Furthermore, previous studies indicate that HP infections are inversely associated with CD (18, 19). However, there is a paucity of studies from Ethiopian testing on the incidence of CDA and associations with LMTB and HP. The aim of the present study was to provide new insight into the incidence of CD and its associations with LMTB and HP infection in Ethiopian children.

## Materials and methods

### Study population

**Figure 1:**
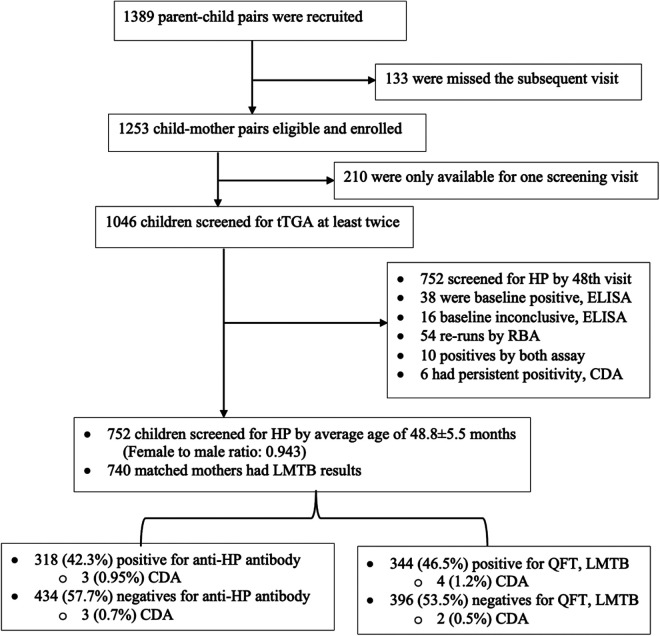
Flow chart of study participants.

The Traditional Ethiopian Food (TEF) project is a longitudinal, observational prospective screening study that follows up a birth cohort at three health facilities in Adama, Oromia, Ethiopia's largest regional state. The purpose is to assess the incidence and prevalence of CD study in relation to genetic and environmental factors, e.g., dietary patterns, nutritional conditions, and exposure to infectious diseases. As of April in 2018, 1,389 participants were recruited for a 4-year follow-up. Annual screening for CD began from 2 years of age (Figure 1). Baseline data were collected during first recruitments for female participants and after delivery by 6 weeks for infants and then followed up at 9, 18, 24, 36, and 48 months for CD. At each visit, trained clinical nurses filled out a questionnaire regarding the participant's feeding practices, clinical information, medications, and growth metrics provided by the legal guardian as previously described (Supplementary Table S1) (20, 21). As of June 2022, 1,256 (90.4%) children had been screened at least once for tTGA using a commercial ELISA, of whom 1,046 (83.4%) had at least two serological tTGA measurements at median age of 49.3 (interquartile range, 47.5–53.3) months. Among the 1,046 recruited children, 752 children had mothers who had been screened for LMTB findings and children themselves screened for HP at a mean age of 49.3 ± 5.3 months.

### Screening for CDA

Baseline screening for CDA started at 2 years of age by measuring IgA-neoepitopes against tTGA as previously described (AESKULISA tTg-A New generation, AESKU DIAGNOSTICS GmbH & Co. KG Mikroforum Ring 2 55234 Wendelsheim, Germany) (22–24). Antibody levels were expressed in U/ml. The cutoff level chosen for the present study was 18 U/ml for IgA anti-tTGA according to receiver operating characteristic curves. A range between 12 and 18 U/ml was considered as the borderline positivity region. Children who were tTGA-positive at baseline were retested for both IgA-tTGA and IgG-tTGA using radioligand-binding assays (RBAs) as previously described (25). tTGA levels were expressed in U/ml, calculated from standard curves. Samples above the highest calibrator range were diluted as appropriate and reassayed. Children who were persistent-confirmed tTGA-positive in two consecutive samples were defined as having CDA and referred to a pediatrician for clinical follow-up (26). The decision whether to refer the child to the tertiary hospital for an upper endoscopy and intestinal biopsy to confirm diagnosis of CD was taken by the pediatrician.

### Screening for LMTB and HP infections

Data on LMTB and related sociodemographic factors were collected during prenatal care and after delivery from mothers in the integrated Pregnancy-TB cohort. LMTB was confirmed by measuring IFN-*γ* using ELISA according to the QFT-Plus protocol (27). The present analysis included 752 children for data on the colonization of HP and mother–child pairs for LMTB exposure. HP infection was examined in the leftover serum samples from the birth cohort that were maintained in the repository. All serum samples collected at an average age of 4 years were analyzed for anti-HP antibody by using the commercially available ELISA (IBL International GmbH, Hamburg, Germany) method as previously described (28).

### Statistical analysis

The data were analyzed using a statistical software package for Social Science, IBM SPSS, version 27. Descriptive statistics, namely, mean, percentages, frequency, and standard deviation, were calculated. The distribution of the parameters was evaluated using the Shapiro–Wilk test. Student's *t* test was used for comparison of two groups with a normal distribution; the Mann–Whitney *U* test was used for comparison of two groups with no normally distributed parameters. The chi-square test was used for comparison of categorical data. The binary logistic regression analysis was used to see the relation between dependent variables and independent variables. Spearman's and Fisher's correlation analyses were used to examine the correlations between metric variables. A *p*-value <0.05 and a width of 95% CI was considered to be statistically significant.

## Results

### Sociodemographic characteristics of the study population

Of the 1,389 mother–child pairs who were invited to take part in the 4-year follow-up, 1,256 underwent at least one IgA-tTGA test, of whom 1,046 were tested at least twice starting at two years of age. Of the 1,046 children, 752 were tested for HP*.* Of these, 740 matched mothers had been checked for LMTB findings and had complete data for analysis were included. The mean follow-up was 50.0 (IQR, 47.5–53.3) months (Table 2).

### Incidence of CDA

A total of 2,433 serum samples from 1,046 children were assessed for IgA-tTGA ELISA. Of the 1,046 (3.6%) children, 38 tested baseline positive, while 16 (1.5%) were borderline positive. Ten children were confirmed to be tTGA-positive with RBA and six of those children were persistent-confirmed tTGA-positive. Detailed information about the six children with CDA is given in Supplementary Table S2. Three children were identified at 24 months of age and the remaining three at 36 months, with an equal number of boys and girls affected by CDA (1:1, *p* = 0.401) (Table 1). The prevalence of CDA estimated at 0.6% (6/1046) (95% CI 0.23–1.28) and incidence of CDA by age four years was 1.2 per 1,000 person-years. One CDA child had an IgA-tTG level >10 times the upper limit of normal (ULN) supporting the diagnosis of CD. The other five CDA children had persistent elevated IgA-tTG levels that could indicate CD but were yet to be confirmed histologically. Five of the six CDA children carried any of the HLA-DQ2 and/or DQ8 haplotypes, with the exception of one child who had a sequencing failure during the HLA genotyping (Table 1). Most children were asymptomatic and there was no difference in anthropometrics between the two groups (Tables 2, 3).

**Table 1:** Observed factors in children with and without CDA.

Variable		CDA+ (*n* = 6)	CDA− (*n* = 1040)	Odds ratio	*P*-value
Child sex	Females	3	507	1.05	0.952
	Males	3	533		
HLA-DQ2 or DQ8	No	0	364		
	Yes	5	476	8.3	0.150
Gluten-rich meals/day	<2 per day	2	165		
	>2 per day	4	875	0.38	0.266
Medication history	No	4	841		
	Yes	2	143	2.9	0.219
Symptoms^a^	No	6	967		
	Yes	0	73	1.02	0.989
*Helicobacter pylori*	NEG	3	431		
	POS	3	315	1.4	0.704
LMTB	NEG	2	394		
	POS	4	340	2.3	0.337
Family size	<4 members	5	738		
	≥4 members	1	302	0.49	0.514
Gluten-free diet, teff	<2 per day	2	743	0.202	0.066
	>2 per day	4	297		

CDA, celiac disease autoimmunity; LMTB, latent Mycobacterium tuberculosis; > or <2 per day: consumption of gluten over/below two times per day.

^a^ Diarrhea (n = 36), constipation (n = 10), cutaneous (n = 9), and others (n = 18).

**Table 2:** Characteristics of study population stratified by HP (*n* = 752) and LMTB (*n* = 740).

Variable	Study participants, *N* (%)					
	HP-positive 318 (42.3%)	HP-negative 434 (57.7%)	*p*-value	TB-exposed 344 (46.5%)	TB-unexposed 396 (53.5%)	*p*-value
Mean age, m (SD)	50.03 (4.88)	48.8 (5.5)	0.002*	50.03 (4.88)	48.8 (5.5)	0.002*
Male, sex, 387 (51.5%)	167 (52.5)	220 (50.7)	0.621	179 (52.0)	203 (48.0)	0.883
Family size, ≥4 members, 471 (62.6%)	205 (64.5)	266 (61.3)	0.374	239 (69.5)	226 (57.1)	0.001*
Houses with multiple room, 371 (53.9%)	156 (53.8)	215 (54.0)	0.953	173 (56.2)	194 (52.4)	0.331
Delivery mode CS, 94 (13.3%)	33 (11.2)	61 (14.8)	0.164	39 (12.3)	54 (14.3)	0.435
Nonexclusive breastfeeding by 6 weeks, 37 (5.6%)	13 (4.8)	25 (6.4)	0.381	15 (5.0)	22 (6.2)	0.513
Breastfeeding up to 18 months, yes, 360 (52.6%)	146 (49.8)	214 (54.7)	0.204	180 (58.4)	173 (47.5)	0.005*
Teff feeding history, yes, 258 (38.5%)	100 (36.0)	158 (40.3)	0.256	126 (19.1)	129 (19.5)	0.369
Gluten-rich cereal feeding, yes, 257 (38.4%)	100 (36.0)	157 (40.1)	0.285	129 (40.3)	129 (36.9)	0.416
Child GI symptoms, yes, 20 (2.7%)	11 (3.5)	9 (2.1)	0.243	6 (1.7)	14 (3.5)	0.134
Child medication history, yes, 28 (3.8%)	15 (4.8)	13 (3.1)	0.240	14 (4.3)	14 (3.6)	0.656
CDA status, positive, 6 (0.6%)	3 (0.95)	3 (0.7)	0.702	4 (1.2)	2 (0.5)	0.424
TB exposure, maternal QFT, positive, 344 (46.5%)	147 (47.4)	197 (45.8)	0.666	—	—	—
Mean weight, kg (SD)	15.87 (2.07)	15.56 (2.21)	0.051	15.56 (2.22)	15.79 (2.07)	0.146
Mean height, cm (SD)	101.73 (5.32)	101.50 (6.21)	0.595	101.55 (6.46)	101.68 (5.23)	0.763

*p value < 0.05.

SD, standard deviation; HP, Helicobacter pylori; CDA, celiac disease autoimmunity; QFT, QuantiFERON test; GI, gastrointestinal; LMTB, latent Mycobacterium tuberculosis; CS, cesarean section; NS, not specified.

**Table 3:** Anthropometrics of children with and without CDA by age.

Metric	By 24 months			By 36 months			By 48 months		
	CDA	No CDA	*P*-value	CDA	No CDA	*p*-value	CDA	No CDA	*p*-value
WHZ (mean, SD)	−0.57 (1.8)	0.07 (1.64)	0.502	−0.36 (0.70)	−0.07 (1.4)	0.644	−0.097 (1.3)	−0.22 (1.1)	0.785
HAZ (mean, SD)	0.23 (0.7)	0.21 (1.8)	0.985	−0.67 (0.9)	−0.61 (1.3)	0.918	−0.86 (1.6)	−0.47 (1.2)	0.429
WAZ (mean, SD)	−0.21 (1.1)	0.21 (1.3)	0.577	−0.62 (0.6)	−0.39 (1.2)	0.669	−1.16 (1.3)	−0.42 (1.0)	0.072
BAZ (mean, SD)	−0.59 (1.9)	0.13 (2.2)	0.572	−0.31 (0.80)	0.001 (1.7)	0.685	−0.89 (0.5)	−0.18 (1.1)	0.115
Mean weight (SD)	11.4 (1.5)	12.3 (1.9)	0.413	13.6 (0.96)	14.1 (2.21)	0.614	14.4 (1.7)	16.0 (2.2)	0.076
Mean height (SD)	86.8 (2.3)	87.3 (5.8)	0.881	95.2 (4.38)	95.0 (5.37)	0.934	100.6 (4.8)	102.7 (5.5)	0.351

SD, standard deviation; CDA, celiac disease autoimmunity; WHZ, weight- for- height z score; HAZ, height-for-age z score; WAZ, weight -for- age z score; BAZ, body mass index for age z score.

### Associations of LMTB and HP with incidence of CDA

Complete LMTB-related data was obtained from 740 mothers whose children had also been tested for anti-HP antibodies. Of these, 344 (46.5%) had LMTB and found in 4/6 (66.7%) mothers to CDA children compared with 340/734 (46.3%) mothers of children without CDA (*p* = 0.424) (Table 1). Among the 752 children of mothers with LMTB also screened for HP at a mean age of 50.0 ± 4.9 months, 318/752 (42.3%) were HP-positive (Table 2). Three of the six children with CDA (50.0%) were HP-positive compared with 315/746 (42.2%) without CDA (*p* = 0.702).

### Diet and anthropometric characteristics of the study participants

Overall, 4.4%, 9.2%, and 5.9% of the children in this study were wasted, stunted, and underweight, respectively, by 4 years of age. The majority of children were fed with teff and gluten-rich, wheat, or barley grains on a regular basis. Two or more serving portions per day of teff or gluten-rich foods did not increase the risk of developing CDA. At the 6-week study visit, the majority (94%) had been exclusively breastfed. Up until the age of 18 months, nearly half of the mothers breastfed their children, but history of breastfeeding was not found to be associated with CDA.

## Discussion

This prospective birth cohort study found a 0.6% prevalence of CDA in the screening of Ethiopian children up to 4 years of age and a corresponding incidence of 1.2 per 1,000 person-years. This is lower than the pooled global seroprevalence of CDA, which varied from 1.1% (Africa) to 1.4% (Oceania and North America) (29), but corresponded to the prevalence of CD reported from Egypt (0.5%) (30), Libya (0.8%) (31), Tunisia (0.6%) (32), and Western Europe (0.6%) (29). This finding of importance further refutes the devoid of CD in sub-Saharan Africa along with case reports from Sudan (33), Djibouti (34), and Ethiopia (14). In a previous study, we estimated the prevalence of tTGA positivity in Ethiopian adult women to be 0.05% (15). Compared to the adult population, the CDA prevalence in the present pediatric cohort increased 12 times. The discrepancy between the two cohorts cannot be explained by methodological reasons since the same assays were used to assess tTGA. We speculate that the reasons for this increase in the prevalence between the two cohorts can be attributed to changes in exposure environmental factors such as infections, adoption of a more western lifestyle, increase of wheat consumption, and a rise in exports of teff injera (35, 36).

First, we noticed during the follow-up screening that many children had transient tTGA levels and seroconverted to tTGA negativity. Although the reason some children seroconvert to tTGA negativity while others progress to developing CD remains unknown, the phenomenon of fluctuating tTGA levels has been described in several previous longitudinal birth cohort studies (37, 38). In this population, we anticipated that most parents in the research area often use injera or flat bread as dietary bases. Teff is an important food grain in Ethiopia, a cereal that lacks T-cell stimulatory peptides and is gluten-free (39). It seems that eating foods free of gluten, such as teff, reduces tTGA levels. This is supported by research findings on the extent of tTGA after a gluten-free diet (40).

Alternatively, falsely tTGA-positive results may have occurred due to the poor diagnostic performance of the assays. A sequential testing approach was employed for initial screening with the highly sensitive ELISA assay ASKULISA, followed by the more specific RBA, a previously used assay in several screenings for CD. It was believed that ELISA tests would be more easily accessible and fairly priced in the setting where the research was conducted and will be used in future clinical screenings. Our findings indicate that ELISA identified more positive results than RBA, which is in line with our previous studies demonstrating that ELISA had lower specificity but equivalent sensitivity to RBA (22, 23, 41). In addition, we found that HLA-DQ2 and/or DQ8 were presented in all CDA-positive children, with an exception of one child whose sequencing phase failed during the genetic analysis. It was shown that the risk of CD for children carrying HLA risk genotypes is eight times higher than children with nonrisk HLA genotypes (42).

Second, we tested if the incidence of CDA was associated with two silent chronic infections—LMTB and HP. Although the exposure of LMTB among Ethiopian mother–child pairs was alarmingly high, there was no association found with CDA incidence in this cohort. This is in contrast to a previous study that found noticeably higher prevalence rates of LMTB in CD patients (43, 44). Autoimmune disorders are more common in groups that have been exposed to tuberculosis and developed resistance to it, and less common in populations that have not (45). In addition, we found that 42.3% of Ethiopian children were HP-positive by 4 years of age. Earlier research showed that the prevalence of HP colonization in the Ethiopian population has decreased from 64.4% to 42.9% between 1990 and 2017 (46). Despite this high but decreasing prevalence, we found no association with CDA incidence in this cohort. However, this contrasts to previous research showing inverse association between lower incidence of infections and increased risk of autoimmune disease (47, 48). The finding is also in contrast to previous studies indicating that HP infection would protect against CD (19). Reasons for inconsistent results between this current study and previous studies might be due to statistical power, sociodemographic differences, research design, diagnostic techniques, and the geographic settings. Moreover, compared to Sweden, the prevalence of HP infection is higher in Ethiopian children, whereas as the opposite seems to be true for CD (49).

The strength is the prospective study design of Ethiopian children enrolled from the general population to a 4-year longitudinal follow-up. This allows for monitoring children from birth and collecting information prior to the onset of CDA without recall bias. Furthermore, the strict definition of CDA, i.e., being persistent tTGA-positive in two consecutive samples collected 1 year apart, in addition to carrying HLA risk genotypes from previous HLA genotyping of the entire cohort (20), most likely excluded many false tTGA-positive individuals, In addition, it allowed us to conclude that the lower incidence of CDA in the present cohort was not dependent on a skewed HLA population. Another strength was the unbiased data collected from mother–child pairs of two highly prevalent chronic infections. However, this study did have a few limitations. For instance, diet-related data were qualitative in nature since data collection was mainly based on parent feedback and not free from recall bias. Since the study was conducted in only a single city of the region of Oromia, it was not representative of the entire region of the country. Another limitation was lack of testing for endomysial autoantibodies and limited access to upper endoscopy for intestinal biopsy to confirm the diagnosis of CD according to the ESPGHAN criteria in children with CDA, which is a common problem in many developing countries like the situation in Ethiopia. Finally, TB exposure in children was based on the information recalled by their mothers either before or after birth.

## Conclusion

The incidence of CDA in Ethiopian children from the Oromia region appears to be higher than that of adults but lower than the pooled global incidence. Exposures to LMTB or childhood HP infections are not associated with incidence of CDA at age 4 years. Therefore, the reason for this increase in prevalence remains to be explored. It is recommended that the current study be expanded to include a larger geographic area of the country, allowing for the regional mapping of CD.

## Data Availability

The original contributions presented in the study are included in the article/Supplementary Material, further inquiries can be directed to the corresponding author.
