# Supplementary material for: Incidence of celiac disease autoimmunity and associations with maternal tuberculosis and pediatric Helicobacter pylori infections in 4-year-old Ethiopian children followed up in an HLA genotyped birth cohort
Source: Front Pediatr. 2022 Oct 26;10:999287. doi: 10.3389/fped.2022.999287 (PMC9644195; doi:10.3389/fped.2022.999287)
Supplement: Supplementary file 1 [file Table1.pdf]

Supplemental table. Levels of IgA-tTG in children with celiac disease autoimmunity.

No.	Sex	HLA	Age 24th months		Age 36th months		Age 48th months	
			IgA-tTG level ELISA	IgA-tTG level RBA	IgA-tTG level ELISA	IgA-tTG level RBA	IgA-tTG level ELISA	IgA-tTG level RBA
#1	female	DQ2.3/X	68.9	20.2	73.6	24.6	67.69	NA
#2	male	DQ2.5trans	0.00	NA	762.91	166.5	77.8	NA
#3	male	NA	139	54.5	95.55	31.9	116.6	NA
#4	female	DQ2.5trans	380.9	22.1	55.8	NA	0.00	NA
#5	male	DQ2.5/X	0.0	NA	67.5	78.1	57.4	NA
#6	female	DQ2.3/DQ8	70.4	10.5	98.8	NA	3.66	NA

Foot note: Cut off level for IgA-tTG ELISA is 18 IU/ml; Cut-off level for IgA-tTG RBA is 4 U/ml. NA; Not available.
