# Supplementary material for: Incidence of celiac disease autoimmunity and associations with maternal tuberculosis and pediatric Helicobacter pylori infections in 4-year-old Ethiopian children followed up in an HLA genotyped birth cohort
Source: Front Pediatr. 2022 Oct 26;10:999287. doi: 10.3389/fped.2022.999287 (PMC9644195; doi:10.3389/fped.2022.999287)
Supplement: Supplementary file 2 [file Datasheet1.pdf]

Study code:

# The Child
# Celiac Disease
# Cohort Study

## Child Follow- 9th months and onwards

| | |
|---|---|
| Study visit number | |
| Date of visit | |
| Next appointment date | |
| Study site | |
| Investigator *(Name & Signature)* | |

| **History and Feeding Behavior** | |
|---|---|
| **Caretaker?** | ☐ Mother ☐ Father ☐ Other relative<br>☐ Orphanage ☐ Unrelated persons |
| **Is the mother alive?** | ☐ Yes ☐ No ☐ Unknown<br>If no, indicate date of death_______________<br>Cause of death___________________________ |

| **Infant feeding** | |
|---|---|
| **Is your child currently being breast feed?** | ☐ Yes ☐ No |
| *If no, breastfeeding was ended at the age of:* | |
| *If yes, type of feeding?* | ☐ Exclusive breastfeeding<br>☐ Mixed feeding<br>☐ Exclusive bottle feeding |
| **Has your baby received any other foods or infant formula than breast milk?** | ☐ Yes ☐ No |

| **If yes which of the following foods?** | **At what age in months?** | **If yes which of the following foods?** | **At what age in months?** |
|---|---|---|---|
| Fruits or berries | | Teff * | |
| Potatoes | | Corn (Maize) | |
| Yams, Sweet potatoes | | Oats | |
| Nuts and seeds | | Beef | |
| Cabbages | | Poultry | |
| Root vegetables. - *carrots, turnips, parsnip* | | Lamb | |
| | | Goat | |

Study code:

| | | | |
|---|---|---|---|
| Fruit vegetables *tomatoes, cucumber, squash, spinach* | | Fish and other seafood | |
| Legumes | | Egg | |
| Other vegetables | | Milk products | |
| Rice | | Cheese | |
| Wheat | | cow milk | |
| Barley | | Soy milk | |
| Lentils | | chickpea | |
| Enset | | Goat/camel/sheep milk | |
| Buckwheat, Millet | | Other commercially readymade food, Specify ____________________ | |
| Sorghum | | | |

| | |
|---|---|
| Has your child eaten a gluten-free diet (such as foods based on teff)? | ☐ Never<br>☐ Once per day<br>☐ >= 2 per day<br>☐ Once a week<br>☐ 1-3 times a month<br>☐ Other, specify |
| Has your child eaten food containing wheat, barley, or millet? | ☐ Never<br>☐ Once per day<br>☐ >= 2 per day<br>☐ Once a week<br>☐ 1-3 times a month<br>☐ Other, specify |

## Symptoms

| | |
|---|---|
| **Fever**? | ☐ Yes ☐ No |
| *If yes, duration of fever?* | ☐ < 2 days ☐ 2 days – 1 week<br>☐ 1 week – 1 month ☐ > 1 month ☐ unknown |
| **Feeding problems**? | ☐ Yes ☐ No<br>☐ If yes, specify---------------------------------------- |
| **Loss of appetite?** | ☐ Yes ☐ No |
| *If yes, duration of loss of appetite?* | ☐ < 2 days ☐ 2 days – 1 week<br>☐ 1 week – 1 month ☐ > 1 month ☐ unknown |
| **Failure to thrive** | ☐ Yes ☐ No |
| Cough | ☐ Yes ☐ No |
| *If yes, duration of cough?* | ☐ < 2 days ☐ 2 days – 1 week<br>☐ 1 week – 1 month ☐ > 1 month |

Study code: 

| | ☐ Unknown | |
|---|---|---|
| **Seizure** | ☐ Yes | ☐ No |
| **Diarrhea in previous 4 weeks?** | ☐ Yes | ☐ No |
| **Abdominal pain in previous 4 weeks?** | ☐ Yes | ☐ No |
| **Headache in previous 4 weeks?** | ☐ Yes | ☐ No |
| Noticed palpable lymph nodes? | ☐ Yes | ☐ No |

**Celiac disease associated symptoms**

| **Does your child have any of the following symptoms or findings since previous visit:** | | Yes | No |
|---|---|---|---|
| | Diarrhea? | | |
| | Vomiting/nausea? | | |
| | Constipation? | | |
| | Abdominal distension? | | |
| | Failure to thrive? | | |
| | Poor appetite? | | |
| | Muscle wasting? | | |
| | Gas/flatulence? | | |
| | Retarded length growth? | | |
| | Weight loss? | | |
| | Irritability? | | |
| | Pale, foul-smelling, or fatty stool? | | |
| | Dermatitis herpetiformis (itchy skin rash) | | |
| | Malabsorption of ingested fat (steatorrhea) | | |

**Physical Examination**

| | | |
|---|---|---|
| Length/height (cm) | ……………………………… | |
| Weight (kg) | ……………………………… | |
| MUAC (cm) | ……………………………… | |
| Head circumference | ……………………………… | |
| Conjunctiva pallor | ☐ Yes | ☐ No |
| Abdominal distension | ☐ Yes | ☐ No |
| Abdominal mass | ☐ Yes | ☐ No |
| Peripheral edema | ☐ Yes | ☐ No |
| Neck stiffness | ☐ Yes | ☐ No |
| Neurological deficit | ☐ Yes | ☐ No |
| Skin disorder | ☐ Yes | ☐ No |

Study code: 

| | |
|---|---|
| Bleeding diathesis | ☐ Yes ☐ No |

NAME OF THE INVESTIGATOR: ________________SIGNATURE: ____________DATE: ___________

**Laboratory Request form for Child Celiac Disease Cohort Study**

| | | | | | |
|---|---|---|---|---|---|
| Study code | | Date of current visit | | | |
| | | Date of blood sampling | | | |
| | | Date of Stool sampling | | | |
| Current visit after delivery | | ☐ 9 months after delivery<br>☐ 18 months after delivery<br>☐ 24 months after delivery<br>☐ 36 months after delivery<br>☐ 48 months after delivery | | | |
| Next appointment date | __________ | Study site | _____________ | Investigator | __________ |
| **Laboratory Investigations** | | | | | |
| Type of test | Type of Sample | Result | Performed & reported | Approved by | Comments |
| IgA-tTG serological tests | Blood (Serum) | ☐ Negative<br>☐ Positive<br>☐ Indeterminate | | | |
| HLA genotyping study | DBS | | | | |
| Stool direct microscopic examination | Stool | | | | |
| Stool examination for micro- biota study | Stool | | | | |

Specimen Collected by (name & sign):_________________Date:__________________
Checked by (name & sign):________________________Date:____________________
Approved by (name & sign):_____________________Date:______________________

Study code: 

# The Child Celiac Disease Cohort Study

## Child Follow- 24th months and onwards

| | |
|---|---|
| Child gender | ☐ Female<br>☐ Male |
| Date of visit | |
| Next appointment date | |
| Study site | |
| Current visit | ☐ 24 months after delivery<br>☐ 36 months after delivery<br>☐ 48 months after delivery<br>☐ 60 months after delivery<br>☐ Unscheduled Visit |
| Investigator*(Name & Signature)* | |

**HEALTH/SUPPLEMENTARY QUESTIONS**

| | | | |
|---|---|---|---|
| How many individuals live together in this Household? | | | |
| Is your child DBS was taken in any of your previous visit? | ☐ Yes ☐ No | Check for Log | if "no" include by this visit |
| Was your child sick in the last 15 days? | ☐ Yes ☐ No | If "yes" continue | if "no" skip to the next Q |
| Diarrhea | ☐ Yes ☐ No | | |
| Cough | ☐ Yes ☐ No | | |
| Fever | ☐ Yes ☐ No | | |
| Food malabsorption | ☐ Yes ☐ No | | |
| Other, specify | ☐ Yes ☐ No | | |
| Was your child visit any health institute for medical care in the last six months? | ☐ Yes ☐ No | If "yes" continue | if "no" skip to the next Q |
| If "yes" why visit? | ☐ | | 1=sick; 2=vaccine; 3=check up |
| If "yes" where you look for care? | ☐ | | 1=Health center; 2= private clinic/hospital 3= Public Hospital |
| Was your child took any medication in the last six months? | ☐ Yes ☐ No | | if "no" skip to next Q |

Study code: 

| | | | |
|---|---|---|---|
| Anti-biotic drug, if possible specify | ☐ Yes ☐ No | Specify | |
| Anti-viral drug, if possible specify | ☐ Yes ☐ No | Specify | |
| Anti-helminthic drug, if possible specify | ☐ Yes ☐ No | Specify | |
| Anti-allergic drug, if possible specify | ☐ Yes ☐ No | Specify | |
| Anti-mycobacterium drug, if possible specify | ☐ Yes ☐ No | Specify | |
| Anti-protozoan drug, if possible specify | ☐ Yes ☐ No | Specify | |
| ORS/Zinc | ☐ Yes ☐ No | | |
| Was the amount of food that YOUR CHILD ate yesterday similar to what HE/SHE normally eats? | ☐ Yes ☐ No | | 1= Usual; 2= Less than usual; 3= More than usual |
| What is the main reason IF the amount your child ate yesterday was LESS THAN USUAL? | ☐ | IF" LESS THAN USUAL" | 1=little food available<br>2= out of food<br>3=travelling;<br>4= Sickness;<br>5=sick day;<br>6=Social function;<br>7=stressed; 8 other; 9= unknown |
| What is the main reason IF the amount your child ate yesterday was MORE THAN USUAL? | ☐ | IF" MORE THAN USUAL" | 1=travelling;<br>2=feast/ holiday; 3=On vacation or day off; 4=very hungry<br>other; 7= unknown |
| In the past 2 weeks, has your child taken any vitamin or mineral supplements or PlumpyNut? | ☐ | | |
| Vitamin A | ☐ Yes ☐ No | | |
| Iron | ☐ Yes ☐ No | | |
| Zinc | ☐ Yes ☐ No | | |
| PlumpyNut | ☐ Yes ☐ No | | |
| Other | ☐ Yes ☐ No | | |

Study code:

# 24 Hour Diet Recall

Now I would like to ask you about everything of YOUR CHILD consumed from the time you awoke yesterday until the awaking today. If you are not sure of the time, you can describe from sunrise yesterday to sunrise today.

| **List of Food ingredients/items** | **Time (local time)** | **Occassion** | **Quick List of food Types** (column A) | **Where did you obtain the (FOOD)? Or Source** (Column B) | **Amount of FOOD** **(Describe as; 1=** small; 2= medium **3=** large) or use either: Tea spoon, soup spoon, Chilfa, coffe cup, tea cup, portion, picies… |
|---|---|---|---|---|---|
| 1. Teff<br>2. Wheat<br>3. Rice<br>4. Barley<br>5. Millet<br>6. Corn<br>7. Sorghum<br>8. Enset<br>9. Oats<br>10. Soy milk<br>11. Chickpea<br>12. Potatoes<br>13. Nuts<br>14. Tomato<br>15. Legumes; Lentils, peas, beans..... | | Breakfast | | | |
| | | Brunch | | | |
| | | Lunch | | | |
| | | Snack | | | |
| | | Dinner | | | |
| | | Late night meal | | | |
| | | Fruit | | | |
| | | Other | | | |

**Types of Food (column A)**

1. Injera (sourdough flatbread)
2. Bread
3. Chechebsa (kita fir fir )
4. unleavened breads (*kitta*)
5. Anebabero
6. Porridges (Gonfo)
7. Shiro wat
8. Misir wat
9. Tibs (meat chunks)
10. Kitfo (Ethiopian beef tartare)
11. Doro wat/poultry
12. Salata (Ethiopian salad)
13. Fruits/Juices.

11. Injera Fitfit (Firfir)
12. Fish
13. Gruels (Atmit)
14. Cabbage (Gomen Be siga)
15. Pasta/ Endomi
16. Makoroni
17. Cake
18. Biscute
19. Rice
20. Egg
21. Milk/cheese/Yogurt
22. Kolo
23. Other specify_________

**Source of food (Column B)**

1. Homemade
2. Restaurant/cafeteria/fast food shop/deli
3. Food stall/hawker
4. Supermarket/Food store
5. Workplace tuck shop
6. Day care
7. Friend/relative's home
8. Party/banquet/special event
9. Other specify):_______________________

| | |
|---|---|
| Has your child eaten a gluten-free diet (such as foods based on teff)? | ☐ Never ☐ Once per day<br>☐ >= 2 per days ☐ Once a week<br>☐ 1-3 times a month<br>☐ Other, specify |
| Has your child eaten food containing wheat, barley, or millet? | ☐ Never ☐ Once per day<br>☐ >= 2 per day ☐ Once a week<br>☐ 1-3 times a month<br>☐ Other, specify |

## Celiac disease associated Symptoms

Study code: ________

| Does your child have any of the following symptoms or findings since previous visit: | | Yes | No |
|---|---|---|---|
| | Feeding Problem? | | |
| | Diarrhea? | | |
| | Vomiting/nausea? | | |
| | Constipation? | | |
| | Abdominal distension? | | |
| | Failure to thrive? | | |
| | Chronic diarrhea with or without abdominal pain | | |
| | Physically fatigued or exhausted? | | |
| | Gas/flatulence? | | |
| | Retarded length growth? | | |
| | Weight loss? | | |
| | Irritability? | | |
| | Pale, foul-smelling, or fatty stool? | | |
| | Dermatitis herpetiformis (itchy skin rash) | | |
| | Symptomatic malabsorption | | |

**ANTHROPOMETRY AND PHYSICAL EXAMINATION**

| | |
|---|---|
| Clothing worn ☐ | (0=none,1=very light,2=light, 3=med., 4=heavy) |
| Length/height (cm) | Height1 \|______\| Height2 \|______\| |
| Weight (kg) | Weight1 \|______\| Weight 2 \|______\| |
| MUAC (cm) | MUAC 1 \|______\| MUAC 2 \|______\| |
| Child's MUAC<12 cm? | ☐ Ye ☐ No<br>If "Yes", refer to pediatrician/local nutrition experts. |
| Head circumference | Head circumfe 1 \|____\| Head circumfe2 \|______\| |
| Conjunctiva pallor | ☐ Yes ☐ No |
| Abdominal distension | ☐ Yes ☐ No |
| Abdominal mass | ☐ Yes ☐ No |
| Peripheral edema | ☐ Yes ☐ No |
| Neck stiffness | ☐ Yes ☐ No |
| Neurological deficit | ☐ Yes ☐ No |
| Skin disorder | ☐ Yes ☐ No |
| Bleeding diathesis | ☐ Yes ☐ No |

## Laboratory Request form for Child Celiac Disease Cohort Study

Study code: 

Study code:______________

| Study code | Study visit code | Date of current visit | |
|---|---|---|---|
| | | Blood sampling (plasma/serum) | |
| | | Stool/saliva sampling | |
| **Current visit after delivery** | | ☐ 24 months after delivery ☐ 48 months after delivery<br>☐ 36 months after delivery ☐ 60 months after delivery | |

## Laboratory Investigations

| Type of test | Result | Test Performed by | Remarks |
|---|---|---|---|
| **Stool direct microscopic examination** | | | |
| IgA-tTgA serological Assay result | | | |
| HLA Typing | | | |
| Microbiome Analysis | | | |

Requested by (name & sign):________________Date:_____________________________

Specimen Collected by (name & sign):________________Date:___________________

Approved by (name & sign):___________________________Date:___________________
